# Machine learning assisted designing of non-fullerene electron acceptors: A quest for lower exciton binding energy

**DOI:** 10.1016/j.heliyon.2024.e30473

**Published:** 2024-04-29

**Authors:** Jameel Ahmed Bhutto, Bilal Siddique, Ihab Mohamed Moussa, Mohamed A. El-Sheikh, Zhihua Hu, Guan Yurong

**Affiliations:** a College of Computer Science, Huang Gang Normal University, Huanggang, 438000, China; b Department of Chemistry, Division of Science and Technology, University of Education, Lahore, 54770, Pakistan; c Department of Botany and Microbiology, College of Science, King Saud University, P.O. Box 2455, Riyadh, 11451, Saudi Arabia

**Keywords:** Machine learning, Non-fullerene electron acceptors, Exciton binding energy, Synthetic accessibility

## Abstract

The designing of acceptors materials for the organic solar cells is a hot topic. The normal experimental methods are tedious and expensive for large screening. Machine learning guided exploration is more suitable solution. Bagging regression, random forest regression, gradient boosting regression, and linear regression are trained to predict exciton binding energy. Breaking Retrosynthetically Interesting Chemical Substructures (BRICS) methodology has utilized for designing of new non-fullerene acceptors (NFAs). The predicted values were used to select the designed NFAs. On the selected NFAs, clustering and chemical similarity analyses are also performed. Chemical fingerprints are used for this purpose, and the synthetic accessibility score of the new NFAs is also investigated.30 NFAs have selected with low exciton binding energy values. This approach will allow for the rapid screening of NFAs for organic solar cells. Our proposed framework stands out as a valuable tool for strategically selecting the most effective NFAs for organic solar cells and offers a streamlined approach for material discovery.

## 1 Introduction

Organic solar cells (OSCs) are incredibly flexible and lightweight, which is one of their main advantages. OSCs as opposed to conventional silicon-based solar panels, can be used into a variety of novel applications [1,2]. They provide increased adaptability, ranging from flexible solar panels that adapt to irregular surfaces to wearable solar-powered clothes. These cutting-edge photovoltaic technologies are redefining how we produce electricity and opening the door to a cleaner environment by harnessing the power of organic materials [3,4].

Exciton Binding Energy (Eb) is the energy required to dissociate an exciton, which is a bound state of an electron and a hole, into free charge carriers. Due to relative low dielectric constant of non-fullerene acceptors (NFAs), excitons are produced rather than free charge carriers upon activation by incoming light. The Coulombic forces are used to bind these excitons, which can make it difficult for them to dissociate into free charge carriers. The Eb of an organic material is typically in between 0.1 and 1.0 eV, which is substantially greater than that for perovskites. For organic solar cells, the high Eb causes significant energy loss and geminate recombination. Excessive energy loss and severe geminate recombination are caused by high exciton binding energy (Eb) in organic materials in organic solar cells (OSCs). Geminate recombination occurs when the electron and hole of an exciton recombine before they can be separated and collected as current, which reduces the efficiency of the solar cell [5,6]. Furthermore, because of the high Eb and short exciton lifetime, a bulk heterojunction (BHJ) structure with bicontinuous interpenetrating networks and nanoscale phase separation is needed to facilitate exciton dissociation with the energy level difference, which causes issues with morphology control. As a result, lowering the exciton binding energy is essential for further improving OSC device performance [7,8].

Many methods have been developed to reduce the exciton binding energy of organic compounds. Extensive research is required to develop methods for calculating Eb and study the link between the voltage loss and the Eb. The effects of small-molecule acceptor properties on Eb from the standpoints of side chains, molecular packing, end groups, and fused-ring donor cores are required to explored.

There is a trade-off between reducing the exciton binding energy (Eb) and bandgaps/miscibility. Specifically, reducing Eb may lead to changes in morphology of the active layer that can affect the miscibility of acceptor and donor materials and the proficiency of charge separation. Therefore, it is important to carefully balance the reduction of Eb with other factors that affect the stability and performance of an organic solar cells [9].

The traditional mode of cultivating materials is mainly based on the trial and error [10,11]. The continual characterization and synthesis will continue until properties of the virtual materials reach the goal [12]. This lengthy procedure required large sets of material that are time consuming and expensive. To overwhelm these challenges, the computer-aided methods are used to discover, design and screening of compounds [13]. The computational methods use mathematical algorithms to predict, verify and analyze scientific data, which is used to solve complex scientific problems of the various systems [14,15]. Researchers have concentrated on creating the predictive computer model during the past years to aid in their investigation and resolution of difficult problems [16].

The “fourth model of science” that has drawn attention from all over the world is now being referred to as artificial intelligence (AI) [17]. Machine learning (ML) is a recent research tool which has been considered as the core of AI since the 1980s because of its capability to restructure the existing knowledge and to discover implied relationships [18]. It is the discipline of computer science which uses techniques and algorithms to solve complicated problems that are challenging to comprehend using traditional programming techniques [19]. Machine learning algorithms cultivate a mathematical model from the sample data, which is stated as a training data, to draw conclusion and predictions.

Machine learning has been successfully used to study various properties related to organic solar cells. Naeem et al. searched electron-rich and electron-deficient building blocks through data mining to design new polymers [20]. Machine learning was used to predict the reorganization energy. Basha and colleagues utilized machine learning to forecast the highest occupied molecular orbital (HOMO), lowest unoccupied molecular orbital (LUMO), and maximum absorption wavelength (*λ*_max_) [21]. Additionally, their predictions included the synthetic accessibility of the designed molecules, unveiling insights into the feasibility of the suggested new organic compounds. Katubi et al. presented a novel approach for designing acceptor materials intended for use in organic solar cells [22]. The method involves mining of building blocks from a chemical database and enumerating new libraries of these building blocks. Subsequently, acceptors are designed by employing the identified building blocks, and machine learning is employed to predict the power conversion efficiency of these acceptors. Tufail et al. introduced a comprehensive methodology for the screening of organic semiconductors [23]. More than 40 machine learning (ML) models undergo testing to determine the most effective one. The design of 5000 small molecular acceptors (SMAs) is accomplished through the Breaking Retro-synthetically Interesting Chemical Substructures (BRICS) approach.

In current study, several machine learning models are trained to predict exciton binding energy. Molecular descriptors are computed and used as independent features in the training of different machine learning models. The prediction ability of these models is reasonably high. A large library of non-fullerene acceptors (NFAs) is designed. The synthetic accessibility score of the new NFAs is also investigated. 30 NFAs have selected with low Eb values. The purpose of study is to introduce a framework for the fast and efficient screening of materials for organic solar cells.

## 2 Methodology

### 2.1 ML analysis

The data acquisition is one of the major steps in the ML modeling. The data of exciton binding energy is taken from the already documented study [24]. The collected dataset contained about forty-eight thousand points. RDKit is used to compute many types of molecular descriptors for compounds. Machine learning models are trained using these descriptions. Python packages such as Numpy, Matplotlib, Seaborn, Scikit-leran, Scipy, and Pandas have been imported. These programs are necessary for the analysis and visualization of data. The Pandas module is used to import chemical descriptors and exciton binding energy from a comma-separated values (.CSV) file. Machine learning analysis employs bagging regression, linear regression, gradient boosting regression and random forest regression. Cross-validation is accomplished using scikit-learn's cross_val_score function. Hyperparameters are tuned using the Scikit-Learn GirdSearchCV module.

### 2.2 Compound design and similarity analysis

Breaking Retrosynthetically Interesting Chemical Substructures (BRICS) methodology [25] was utilized for designing of new NFAs. BRICS is used with the RDkit program [26]. A set of electron-rich and electron-deficient were used as input. 50,000 NFAs are generated. Using the previously trained machine learning models, the Eb values of the newly created NFAs were predicted. The predicted values were used to select the designed NFAs. On the selected NFAs, clustering and chemical similarity analyses are also performed. Chemical fingerprints are used for this purpose, and the synthetic accessibility score of the new NFAs is also investigated.

## 3 Results and discussion

### 3.1 Molecular descriptors

**Fig. 1:**
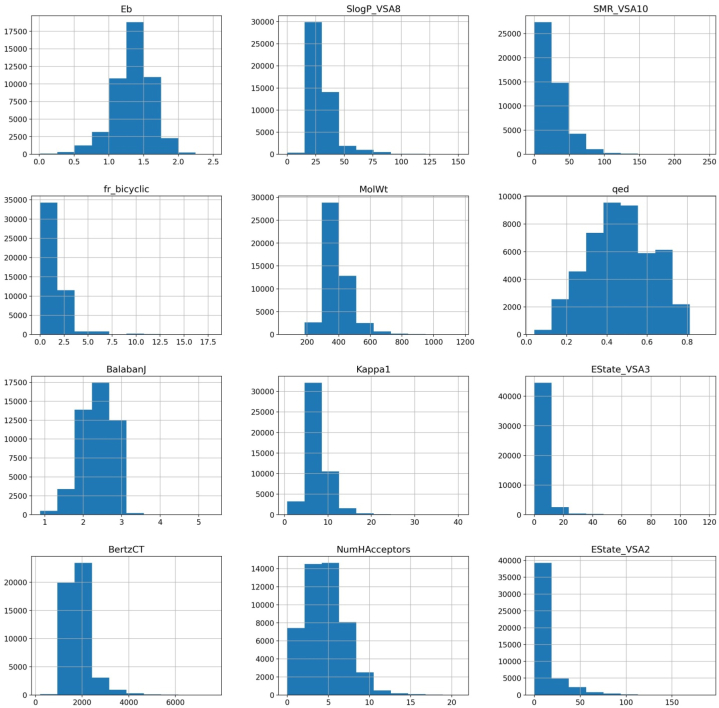
Distribution plot of descriptors (features) and dependent variable (Eb).

Molecular descriptors are mathematical representations of molecular attributes that can be computed using various algorithms [27,28]. The numerical values of molecular descriptors can be used to define (quantitatively) the chemical and physical information of molecules. When building machine learning-based models, the molecular descriptors derived from the molecular structure are used as independent attributes [29,30]. They play a pivotal role in predicting various molecular properties, such as bioactivity, solubility, toxicity, and more, making them indispensable for computational chemistry and virtual screening studies [31,32]. RDKit is an open-source cheminformatics toolkit widely used for generating molecular descriptors. It is employed to comprehensively compute various molecular descriptors, encompassing structural, topological, and physicochemical properties of chemical compounds [33]. These molecular descriptors serve as input features in ML and are essential for developing prediction models. The function of molecules in diverse applications is determined by their chemical structure [34,35]. Molecular descriptors are calculated in order to input the machine learning models [36,37]. In comparison to quantum chemical descriptors, these descriptors are simple and quick to compute [[38], [39], [40], [41]]. The computed descriptors are filters to find best descriptors. The distribution plots for Eb and selected descriptors are given Fig. 1. Majority of compounds have Eb values between 1 and 1.75 eV. Only in case of EState_VSA3, values of are evenly distributed.

### 3.2 Regression analysis

**Fig. 2:**
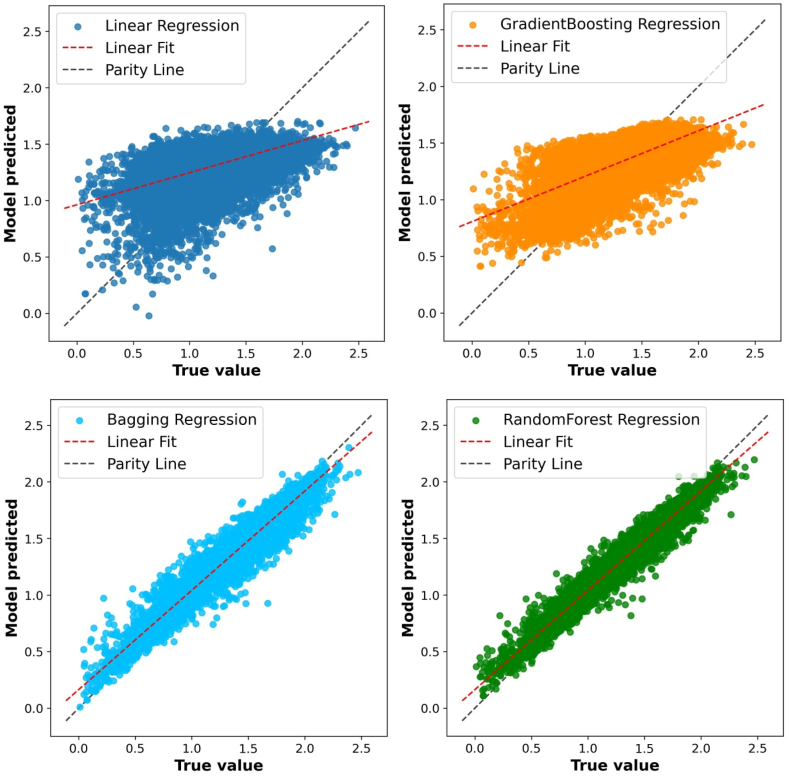
Scatter plot between true and predicted values.

Multiple regressors are utilized to find a good model [42,43]. Cross-validation (CV) is also used; 10-fold CV yielded better results. The performance parameters of many models, such as root mean square error (RMSE) and r-square values are shown in Table 1. It is clear that the random forest regressor is the best. A precise forecast can reduce reliance on costly experimental techniques [[44], [45], [46]]. Fig. 2 depicts scatter plots of the true and predicted values for various models. Random forest regression, bagging regression, gradient boosting regression and random forest regression linear regression have shown R^2^ values of 0.91, 0.85, 0.61, and 0.60 respectively. Linear regression has showed lowest value. Random forest regression is best model.

**Table 1:** R^2^ values for different machine learning models.

Model	R^2^
Linear regression	0.60
Gradient boosting regression	0.61
Bagging regression	0.85
Random forest regression	0.91

### 3.3 Designing new non-fullerene acceptors (NFAs)

When creating NFAs, there are a lot of synthetic choices accessible, whether they are polymers or small molecules [[47], [48], [49]]. Every choice made has an impact on the material's morphology, optoelectronic properties, and ultimate device performance, from substituents to the addition of heteroatoms [[50], [51], [52]]. The availability of customizable organic building blocks can aid in the development of efficient NFAs [[53], [54], [55]]. NFAs with the proper balance of electron-rich and electron-deficient functional units can be used in OSCs. Novel NFAs, in particular, are created utilizing easily synthesized building parts [[56], [57], [58]]. The components are classified into three groups: terminal, core (middle element), and terminal. More than 50,000 NFAs have produced utilizing the Breaking Retrosynthetically Interesting Chemical Substructures (BRICS) approach [25].

The screening of organic semiconductors plays a crucial role in the development of electronic devices and technologies. Screening in the context of organic semiconductors involves evaluating and selecting materials based on their electronic, optical, and structural properties. This process aims to identify compounds that are suitable for organic solar cells.

**Fig. 3:**
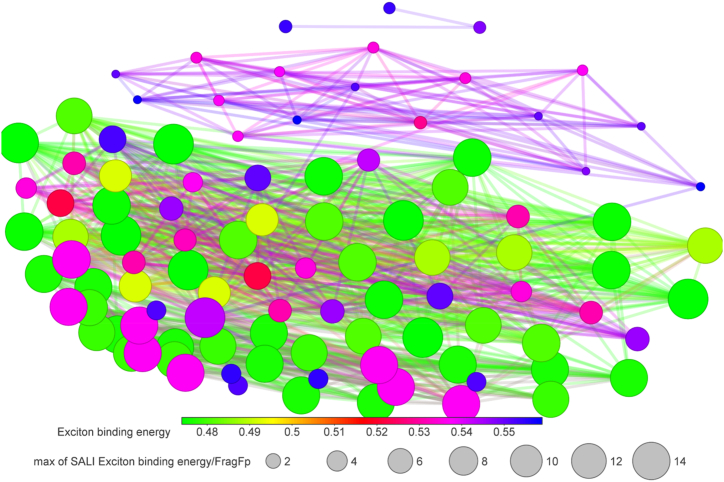
SALI plot for top 100 NFAs with lowest exciton energy.

**Fig. 4:**
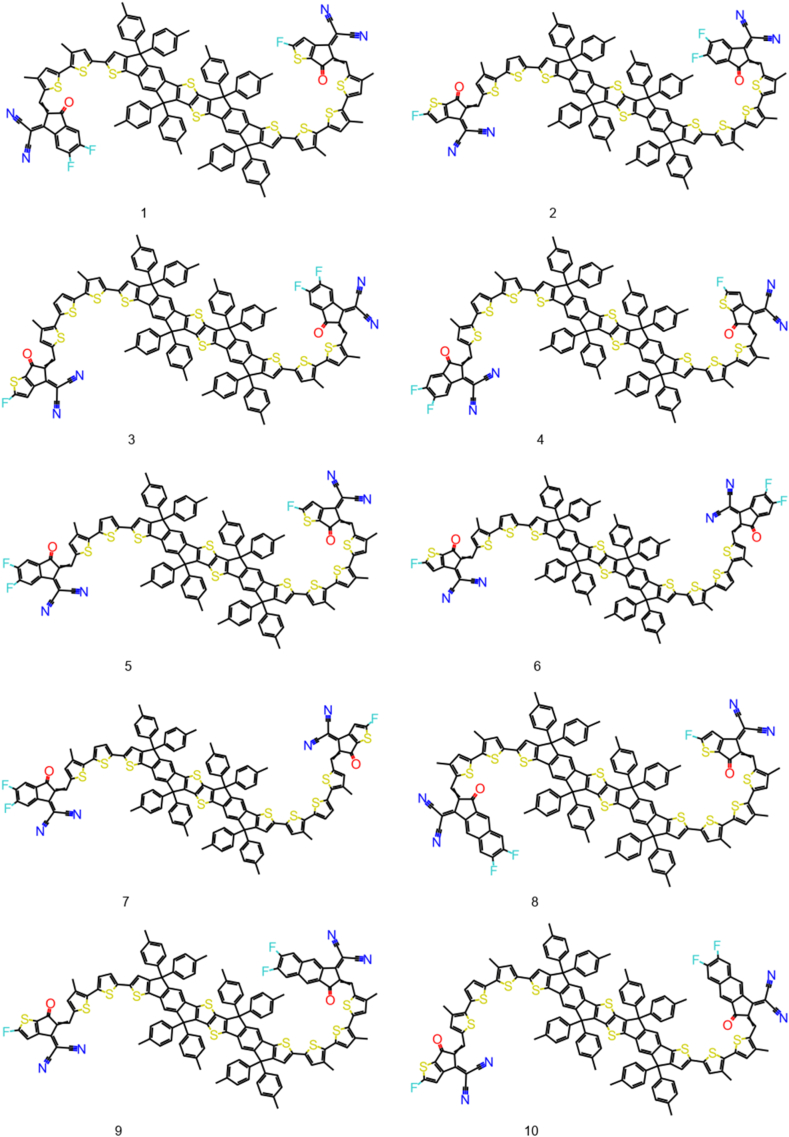
1–10 designed NFAs with lower predicted E_b_.

**Fig. 5:**
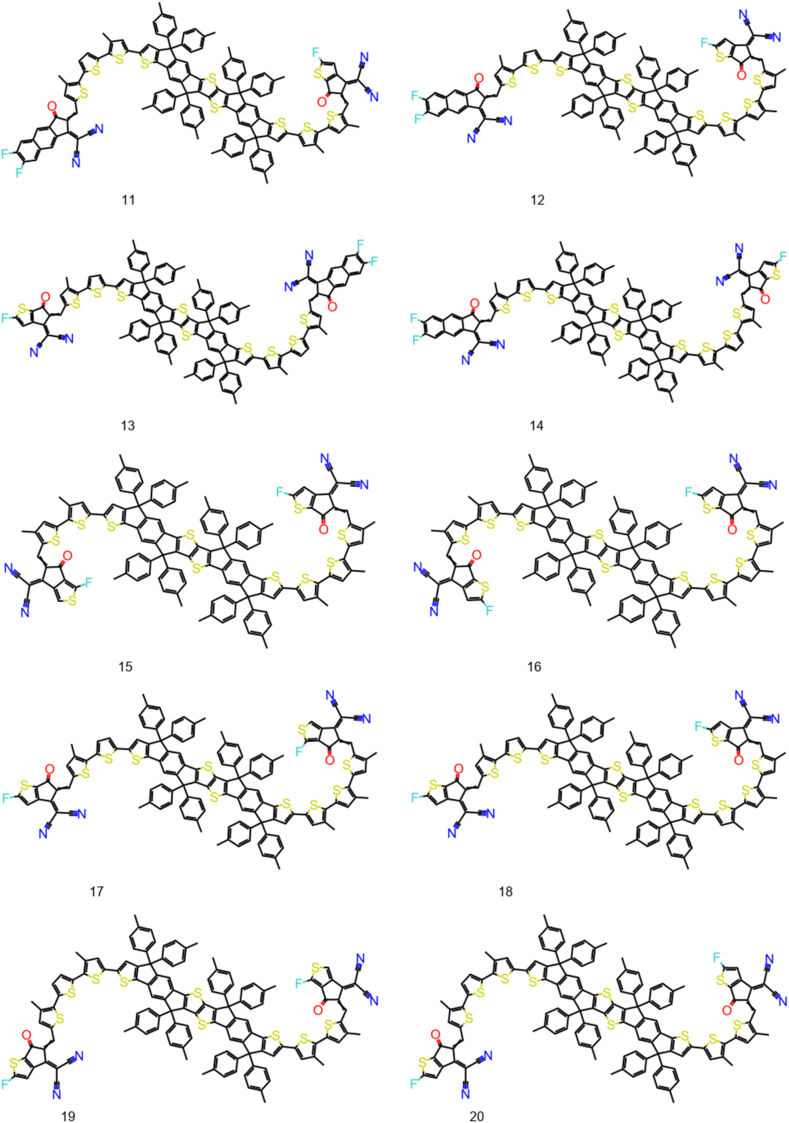
11–20 designed NFAs with lower predicted E_b_.

**Fig. 6:**
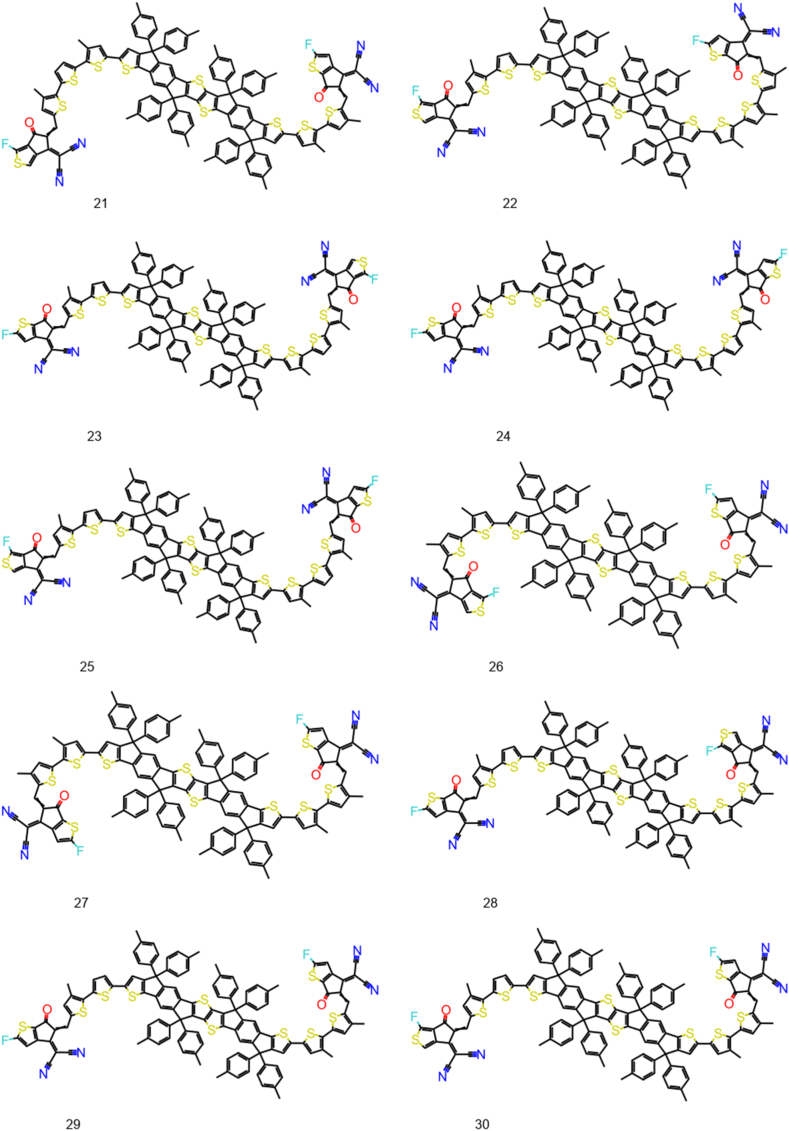
21–30 designed NFAs with lower predicted E_b_.

The Structure Activity Landscape Index (SALI) was introduced by Rajarshi Guha and John Van Drie [59] this is a quantitative assessment of the structure-activity relationship's activity cliffs. Activity cliffs are pairings of structurally related substances that exhibit significant variations in activity, indicating that small changes in structure can have large effect on their activity. SALI is based on the idea that activity cliffs which is a region of high local density in the SAR landscape, surrounded by regions of lower density. SALI calculates the difference in density between a compound and its nearest neighbors in the SAR landscape, and uses this difference to identify activity cliffs. The 100 NFAs with lowest exciton binding energy are used to plot the SALI plot (Fig. 3). There is significant change in exciton binding energy on structural change. The chemical structures of 30 NFA are given in Fig. 4, Fig. 5, Fig. 6. Their exciton binding energy values are given in Table 2. Chemical structures are indicating that selected NFAs have extend conjugation system. It is reported asymmetric structure generally low exciton binding energy due to relative dielectric constant [60].

**Table 2:** The predicted Eb values along with the synthetic accessibility score of the top thirty designed NFAs.

NFAs	Eb (eV)	SA score
1	0.474	7.38
2	0.474	7.4
3	0.474	7.39
4	0.474	7.39
5	0.474	7.4
6	0.474	7.29
7	0.474	7.29
8	0.475	7.54
9	0.475	7.56
10	0.475	7.56
11	0.475	7.56
12	0.475	7.56
13	0.475	7.46
14	0.475	7.46
15	0.476	7.4
16	0.476	7.32
17	0.476	7.42
18	0.476	7.35
19	0.476	7.41
20	0.476	7.34
21	0.476	7.41
22	0.476	7.42
23	0.476	7.32
24	0.476	7.24
25	0.476	7.32
26	0.479	7.17
27	0.479	7.08
28	0.479	7.19
29	0.479	7.11
30	0.479	7.19

### 3.4 Similarity analysis

A comparison of the similarity or dissimilarity between two or more objects is called a similarity analysis [61]. In data analysis, similarity analysis can be used to identify trends, classify items according to their shared characteristics, and provide forecasts. Similarity analysis forms the basis of virtual screening. It is based on the notion that molecules with similar structural characteristics behave in a similar way.

**Fig. 7:**
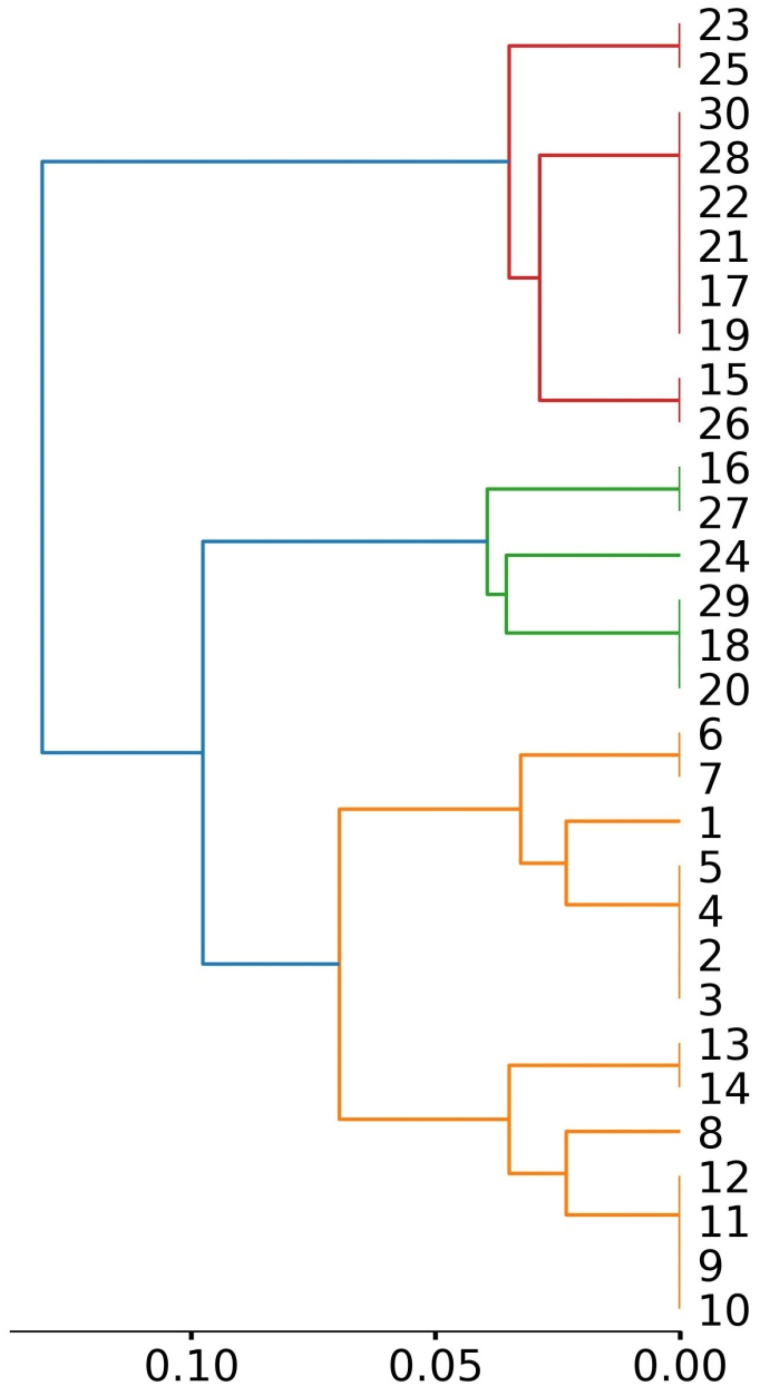
Clustering of monomers based on similarity analysis.

**Fig. 8:**
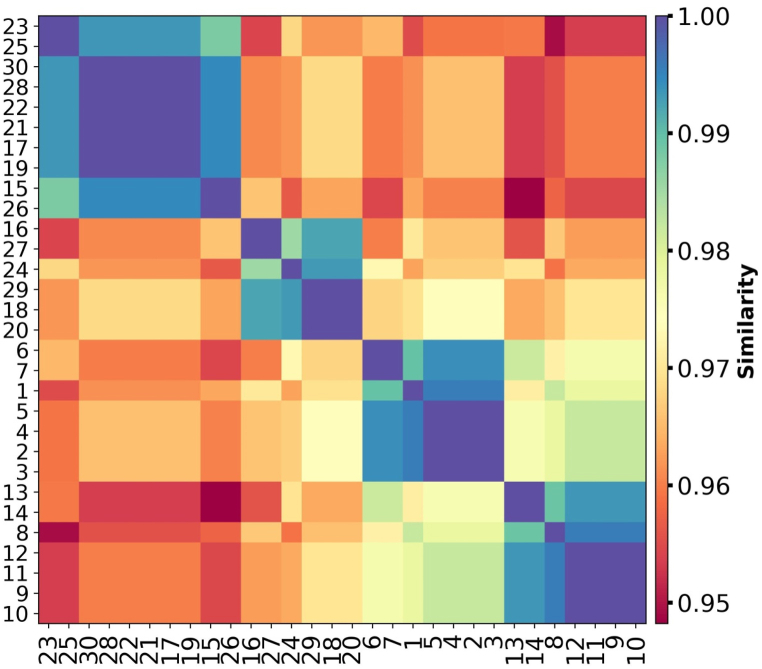
Heatmap of similarity between designed monomers.

In drug discovery, chemical structure-based similarity analysis is a powerful tool for identifying promising molecules. Two molecules with equivalent structures, on the other hand, are likely to perform similarly. The graph in Fig. 7 is dendrogram, which is a tree-like diagram that represents the hierarchical clustering of compounds based on their similarity scores. Each horizontal line in the graph represents a compound, and the vertical lines connecting them indicate the similarity between the compounds. The colors of the lines may represent different clusters or groups of compounds. The figure shows that compounds are structurally versatile. To further validate the similarity of the compounds, we show the similarity of the compounds using a heatmap (Fig. 8). A heatmap is a type of data visualization in which each data point's value is represented graphically by a grid of colored cells. When analyzing large datasets for patterns or trends, heatmaps are frequently utilized, especially in the domains of biology, genetics, and neuroscience. It provides the pairwise similarity between compounds.

The accuracy of our designed framework can be not very high, it is far superior to random screening or designing. Even possibility of finding the few possible applicants by this less expensive way is valuable.

## 4 Conclusions

Gradient boosting regression, random forest regression, linear regression and bagging regression are trained to predict exciton binding energy. Random forest regression has appeared as best model with R^2^ values of 0.91. Breaking Retrosynthetically Interesting Chemical Substructures (BRICS) methodology has used for designing of non-fullerene acceptors (NFAs). 50,000 NFAs are generated. The designed NFAs are shortlisted based on the projected Eb values. 30 NFAs have selected with low Eb values. On the selected NFAs, clustering and chemical similarity analyses are also performed. NFAs are divided in three groups that indicates structural versatility. NFAs the synthetic accessibility score of the new NFAs is also investigated. The synthetic accessibility score helps in synthesis of compounds and similarity analysis helps to analyze the chemical behavior.

## Data availability statement

Data will be made available on request.

## Funding

Researchers Supporting Project number (RSP2024R118), 10.13039/501100002383King Saud University.

## CRediT authorship contribution statement

**Jameel Ahmed Bhutto:** Writing – original draft, Validation, Software, Methodology. **Bilal Siddique:** Validation, Resources, Data curation. **Ihab Mohamed Moussa:** Data curation. **Mohamed A. El-Sheikh:** Formal analysis, Data curation. **Zhihua Hu:** Visualization, Project administration, Investigation, Funding acquisition. **Guan Yurong:** Supervision, Investigation, Conceptualization.

## Declaration of competing interest

The authors declare that they have no known competing financial interests or personal relationships that could have appeared to influence the work reported in this paper.
